# C-Phycocyanin Suppresses the In Vitro Proliferation and Migration of Non-Small-Cell Lung Cancer Cells through Reduction of RIPK1/NF-κB Activity

**DOI:** 10.3390/md17060362

**Published:** 2019-06-18

**Authors:** Shuai Hao, Shuang Li, Jing Wang, Lei Zhao, Yan Yan, Tingting Wu, Jiawen Zhang, Chengtao Wang

**Affiliations:** Beijing Advanced Innovation Center for Food Nutrition and Human Health, Beijing Engineering and Technology Research Center of Food Additives, Beijing Technology and Business University, Beijing 100048, China; lishuangldw@163.com (S.L.); trotwj960@163.com (J.W.); zhaolei@th.btbu.edu.cn (L.Z.); 15128470659@163.com (Y.Y.); m18810529269@163.com (T.W.); zhangjiawen98@outlook.com (J.Z.)

**Keywords:** phycocyanin, non-small-cell lung cancer (NSCLC), proliferation, migration, receptor-interacting serine/threonine-protein kinase 1 (RIPK1), NF-κB

## Abstract

Phycocyanin, derived from Spirulina platensis, is a type of natural antineoplastic marine protein. It is known that phycocyanin exerts anticancer effects on non-small-cell lung cancer (NSCLC) cells, but its underlying mechanism has not been elucidated. Herein, the antitumor function and regulatory mechanism of phycocyanin were investigated in three NSCLC cell lines for the first time: H358, H1650, and LTEP-a2. Cell phenotype experiments suggested that phycocyanin could suppress the survival rate, proliferation, colony formation, and migration abilities, as well as induce apoptosis of NSCLC cells. Subsequently, transcriptome analysis revealed that receptor-interacting serine/threonine-protein kinase 1 (RIPK1) was significantly down-regulated by phycocyanin in the LTEP-a2 cell, which was further validated by qRT-PCR and Western blot analysis in two other cell lines. Interestingly, similar to phycocyanin-treated assays, siRNA knockdown of RIPK1 expression also resulted in growth and migration inhibition of NSCLC cells. Moreover, the activity of NF-κB signaling was also suppressed after silencing RIPK1 expression, indicating that phycocyanin exerted anti-proliferative and anti-migratory function through down-regulating RIPK1/NF-κB activity in NSCLC cells. This study proposes a mechanism of action for phycocyanin involving both NSCLC apoptosis and down regulation of NSCLC genes.

## 1. Introduction

The marine environment is considered as a foremost reservoir of millions of bioactive compounds that have the possibility of being applied in different disciplines, such as medicine, technology, and food [1]. During the past few decades, key compounds substantiating their potential for industrial development as nutritional supplements, functional foods, enzymes, and therapeutic agents have been provided by marine sources [2]. Meanwhile, the antineoplastic effect of marine products has attracted increasing attention. Cytarabine, derived from Caribbean sponge *Tethya crypta*, was discovered as an effective anti-leukemic agent [3]. Trabectedin is isolated from the ascidian *Ecteinascidia turbinate*, an inhabitant of the Mediterranean and Caribbean Seas. It was the first anticancer molecule of marine source that got approval in the European Union to treat reverted incidents of platinum-sensitive ovarian cancer and sarcoma [4]. The discovery of marine products with anticancer abilities could provide a novel method for cancer treatment. 

C-phycocyanin (phycocyanin, C-PC) is one of the major biliproteins existing in Spirulina platensis, as well as a kind of natural marine food colorant [5,6]. Recently, it has attracted increasing attention as a potential functional and healthy food [7]. It has been reported that phycocyanin exerts multiple physiological effects, including anti-inflammatory [8,9], antioxidant [10,11], immunomodulatory [12], and anti-bacterial activities [13], among others. In addition, it also shows good antineoplastic values in different cancer cells, such as breast cancer [14,15], ovarian cancer [16], pancreatic cancer [17], colon cancer [18], and malignant melanoma [19], with no side effects on normal tissue cells [7,14]. Thus, it is necessary to further investigate its potential regulatory mechanism, which might contribute to better development and utilization of phycocyanin.

Lung cancer is one of the most common health threats throughout the world, especially given its high mortality rate [20,21]. Human lung cancer is generally classified into two major categories: small cell lung cancer (SCLC) and non-small-cell lung cancer (NSCLC) [22], of which NSCLC accounts for over 85% of all lung cancer cases, with the characteristics of strong metastasis, low cure rate, and high mortality [23]. Although some new approaches, such as immune-based and targeted therapies, have been introduced in NSCLC treatment [24], the postoperative recurrence rate of NSCLC is still very high, despite most patients being sensitive to the first-line therapy process [25]. Recently, investigators have focused on exploring the effect and underlying mechanism of natural functional products with antineoplastic activities, which would undoubtedly provide valuable information on NSCLC treatment.

The inhibiting function of phycocyanin on NSCLC has been reported in several studies. Bingula et al. reported an anti-proliferative effect of phycocyanin and betaine on NSCLC A549 cells in vitro and in vivo [26]. Li et al. revealed that phycocyanin exerted a synergistic antitumor function with all-trans retinoic acid on A549 cells [27,28]. Baudelet et al. discovered that glaucophyte Cyanophora paradoxa extracts could significantly inhibit the growth of multiple cancer cell lines, including NSCLC A549 cells [29]. In our previous work, we also found an antitumor function of phycocyanin on NSCLC cells [30]. Strikingly, although most studies reported the function of phycocyanin at the phenotypic level, its underlying antineoplastic mechanism in NSCLC still remains unclear. Therefore, further investigation on the regulatory mechanism of phycocyanin on NSCLC cells is critically needed. In this work, the in vitro anticancer effect and underlying mechanism of phycocyanin was explored in three types of NSCLC cell lines (H358, H1650, and LTEP-a2) for the first time, and our results revealed a potential antitumor regulatory approach of phycocyanin, which was expected to lay a theoretical foundation for the future treatment of NSCLC.

## 2. Results

### 2.1. Phycocyanin Inhibits the in Vitro Proliferation of Non-Small-Cell Lung Cancer Cells

**Figure 1:**
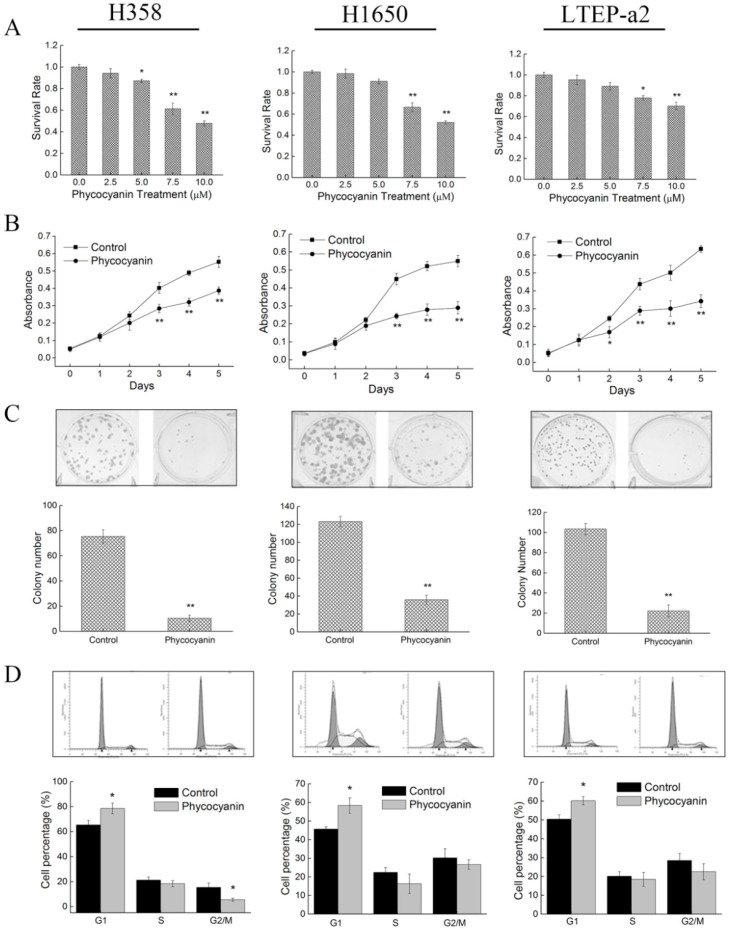
Phycocyanin inhibits the in vitro proliferation of non-small-cell lung cancer (NSCLC) cells. (**A**) Cell survival rate assays on H358, H1650, and LTEP-a2 after different concentrations of phycocyanin treatment (0, 2.5, 5, 7.5, and 10 µM) for 48 h. (**B**) Cell proliferation assays on H358, H1650, and LTEP-a2 after 7.5 µM phycocyanin treatment for 24 h. The proliferation experiment lasted for 5 days. (**C**) Colony formation assay of H358, H1650, and LTEP-a2 after 7.5 µM phycocyanin treatment for 24 h, followed by continuous incubation in fresh medium. The cell colony formation experiment lasted for 12–15 days. (**D**) Cell cycle analyses of H358, H1650, and LTEP-a2 after 7.5 µM phycocyanin treatment for 48 h. Bars represent mean ± SD; * *p* < 0.05; ** *p* < 0.01.

Several studies have indicated the anti-cancer effects of phycocyanin on NSCLC A549 cells [26,27,28,29]. In this present work, to further address and confirm the relationship between phycocyanin and its antineoplastic functions on multiple NSCLC cell lines, we investigated the phenotypic assays on H358, H1650, and LTEP-a2 cells. Phycocyanin in different concentrations (0, 2.5, 5, 7.5, and 10 µM) was used for cell treatment to analyze the viability of NSCLC cells. As shown in Figure 1A, incubation with phycocyanin could dose-dependently reduce the survival rate of H358, H1650, and LTEP-a2 cells. In addition, cell survival rate analysis with high phycocyanin dosage (0, 2.5, 5, 7.5, 10, 15, and 20 µM) is shown in Figure S1, which presents similar results. Strikingly, cell viability was significantly down-regulated when the concentration of phycocyanin was 7.5 and 10 µM. Therefore, we selected 7.5 µM as the treatment dose in the following experiments. Cell proliferation assay showed that phycocyanin could also inhibit the in vitro growth of H358, H1650, and LTEP-a2 cells (Figure 1B). In addition, to investigate the inhibitory role of phycocyanin on the transforming properties of NSCLC cells, we performed a clonogenic assay. As shown in Figure 1C, three types of cells formed well-defined and distinct colonies, and phycocyanin-treated cells displayed significant reductions in colony formation as compared to controls, indicating the potential inhibitory effect of phycocyanin on cell growth and reproductive integrity. Moreover, cell cycle assay was employed to further elucidate the mechanism of phycocyanin-mediated growth inhibition on NSCLC cells (Figure 1D). Results showed that phycocyanin caused significant changes in cell cycle distribution of H358, H1650, and LTEAP-a2 cells. Compared to the control groups, after incubation with 7.5 µM phycocyanin, the proportion of G1 phase cells had significantly increased, suggesting that phycocyanin could cause G1 phase arrest in these three cell lines. Taken together, these results strongly indicated that phycocyanin exerted in vitro anti-proliferative effects on H358, H1650, and LTEAP-a2 NSCLC cells.

### 2.2. Phycocyanin Suppresses the In Vitro Migration of Non-Small-Cell Lung Cancer Cells

**Figure 2:**
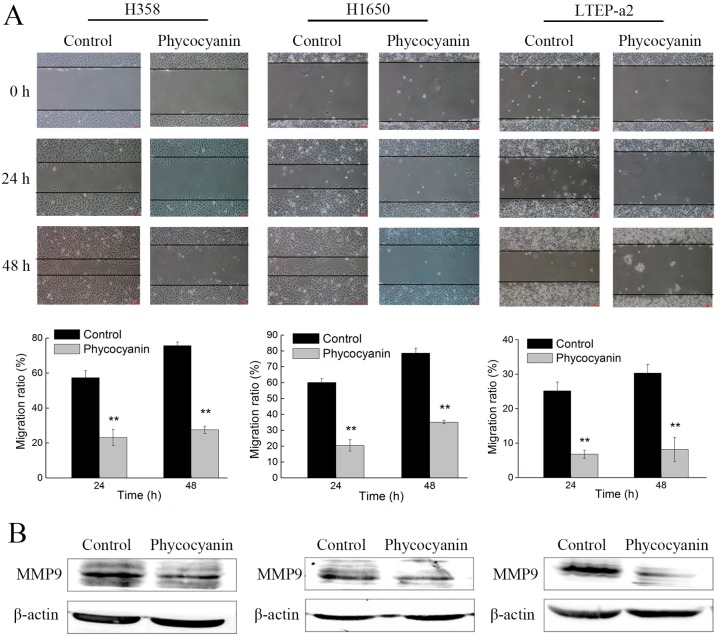
Phycocyanin suppresses the in vitro migration of non-small-cell lung cancer cells. (**A**) The wound-healing assay showed representative effects of phycocyanin (0 and 7.5 µM) on H358, H1650, and LTEP-a2 cell migration at 24 and 48 h. Quantification of wound closure was shown by histogram. Scale bars represent 100 µm. (**B**) Western blot analysis of MMP9 expressions in NSCLC cells after treatment with 7.5 µM phycocyanin for 72 h. MMP9, matrix metalloproteinase-9. Bars represent mean ± SD; * *p* < 0.05; ** *p* < 0.01.

A wound-healing assay was employed to investigate the effect of phycocyanin on cell migration. In this experiment, we cultured cells with medium containing 3% instead of 10% fetal bovine serum (FBS), which could eliminate the contribution of proliferation to the phycocyanin-induced inhibition of cell migration. As shown in Figure 2A, phycocyanin significantly suppressed the migration of H358, H1650, and LTEAP-a2 cells in a time-dependent manner. After treatment with 7.5 µM phycocyanin, the wound closure of H358 cells decreased from 69.72% ± 0.35% to 34.65% ± 0.78%. Similar results were discovered in H1650 and LTEP-a2 cells. Matrix metalloproteinase-9 is a type of gelatinase belonging to the matrix metalloproteinase family, which plays a key role in cancer cell growth and migration due to its ability to degrade extracellular matrix proteins [31]. It was shown that phycocyanin also decreased the expressions of MMP9 in three NSCLC cell lines, which supported the wound-healing analysis (Figure 2B). In addition, we also performed the proliferation and migration analysis of NSCLC using low concentration (2.5 µM) phycocyanin treatment (Figure S2). The results showed that the migration rates of NSCLC cells were also decreased despite the fact that cell proliferation was not affected by 2.5 µM phycocyanin. These results strongly suggested that phycocyanin displayed inhibitory activity on NSCLC cell migration in vitro.

### 2.3. Phycocyanin Induces Apoptosis of Non-Small-Cell Lung Cancer Cells

**Figure 3:**
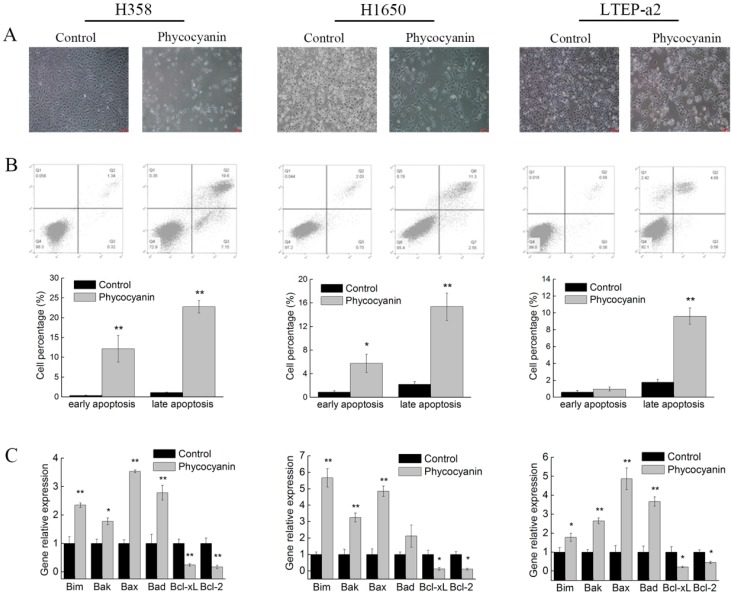
Phycocyanin induces apoptosis of non-small-cell lung cancer cells. (**A**) Cell morphology observation after treatment with phycocyanin (0 and 7.5µM) for 48 h under a light microscope (100×). (**B**) Cell apoptosis analysis using Annexin V-fluorescein isothiocyanate (FITC)/propidium iodide (PI) staining method. Cells were incubated with phycocyanin (0 and 7.5 µM) for 48 h and subjected to apoptosis test. The proportion of early and late apoptotic cells are shown by histogram. (**C**) The qRT-PCR analysis of apoptotic markers in NSCLC cells after phycocyanin (0 and 7.5 µM) treatment for 48 h. Bars represent mean ± SD; * *p* < 0.05; ** *p* < 0.01.

As phycocyanin suppressed proliferation and migration in NSCLC cells, we further explored its pro-apoptotic functions in H358, H1650, and LTEAP-a2 cells. The morphology observation results showed that anomalous changes appeared in cells, some of which became needle-shaped after phycocyanin treatment. The number of cells was also obviously reduced (Figure 3A). Next, the apoptosis of H358, H1650, and LTEP-a2 cells was analyzed by Annexin V-fluorescein isothiocyanate (FITC) and propidium iodide (PI) staining. Figure 3B showed that phycocyanin-treated NSCLC cells demonstrated a significant induction of apoptosis in comparison to untreated cells. The proportion of late apoptotic cells in H358 (22.75% ± 1.57%), H1650 (15.36% ± 2.32%), and LTEP-a2 (9.62% ± 0.98%) significantly increased after incubation with 7.5 µM phycocyanin. To gain a deeper insight into the mechanism of apoptosis induced by phycocyanin, the expressions of classical apoptotic markers were measured by qRT-PCR. As shown in Figure 3C, phycocyanin increased the transcriptional levels of pro-apoptotic genes Bim, Bak, Bax, and Bad, reducing the expressions of Bcl-xL and Bcl-2, two anti-apoptotic genes in H358, H1650, and LTEP-a2 cells. Taken together, the above results suggested that phycocyanin could induce apoptosis in NSCLC cells, which was in accordance with Baudelet’s study [29].

### 2.4. RNA Sequencing (RNA-Seq) Analysis Suggests that RIPK1 is Down-Regulated by Phycocyanin in Non-Small-Cell Lung Cancer Cells

**Figure 4:**
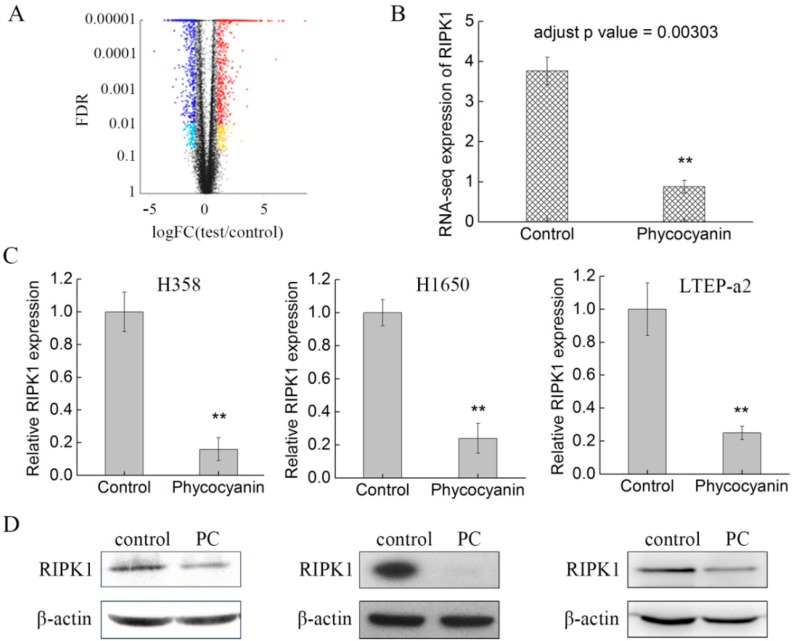
RNA-seq analysis suggests that RIPK1 is down-regulated by phycocyanin in non-small-cell lung cancer cells. (**A**) RNA-seq analysis of differentially expressed genes between control and phycocyanin-treated (0 and 7.5 µM phycocyanin) LTEP-a2 cells. (**B**) RNA-seq analysis of RIPK1 expression in control and phycocyanin-treated (0 and 7.5 µM phycocyanin) LTEP-a2 cells. The duration of phycocyanin treatment was performed 48 h before cell collection and RNA extraction. (**C**) The qRT-PCR analysis of RIPK1 expressions in H358, H1650, and LTEP-a2 cells after phycocyanin treatment (0 and 7.5 µM) for 48 h. (**D**) Western blot analysis of RIPK1 expressions in H358, H1650, and LTEP-a2 cells after phycocyanin treatment (0 and 7.5 µM) for 72 h. RIPK1 = Receptor-interacting serine/threonine-protein kinase 1. Bars represent mean ± SD; * *p* < 0.05; ** *p* < 0.01.

Phycocyanin was proven to exert anti-proliferative and anti-migratory effects on NSCLC H358, H1650, and LTEP-a2 cells. To further explore its anti-cancer mechanism in NSCLC, a high-throughput RNA-seq was employed for the systematic analysis of gene expressions in LTEP-a2 cells after phycocyanin treatment. A comparison at adjusted *p* ≤ 0.05 and log2FC fold change ≥ 1 or ≤ −1 was made to identify the number of DEGs for different groups (Figure 4A). Gene expression analysis showed that 1640 genes were significantly differentially expressed, including 919 down-regulated and 721 up-regulated genes. Strikingly, receptor-interacting serine/threonine-protein kinase 1 (RIPK1) was shown as a significant down-regulated differential protein (adjust p value = 0.00303) in phycocyanin-treated LTEP-a2 cells (Figure 4B). RIPK1 is an essential signaling note in various innate immune signaling pathways, being most extensively studied in the tumor necrosis factor receptor 1 (TNFR1) signaling pathway, which is involved in multiple biological processes, including cell death, cell growth, and tissue homeostasis [32,33]. To validate the RNA-seq results, we examined the expressions of RIPK1 in phycocyanin-treated LTEP-a2, as well as H358 and H1650 cells. As expected, phycocyanin could significantly reduce the transcription and protein levels of RIPK1 in H358, H1650, and LTEP-a2 cells (Figure 4C,D). Taken together, these results indicated that RIPK1 was likely to be involved in the phycocyanin-mediated antineoplastic process in NSCLC cells.

### 2.5. Knockdown of RIPK1 Expression Inhibits the In Vitro Proliferation of Non-Small-Cell Lung Cancer Cell

**Figure 5:**
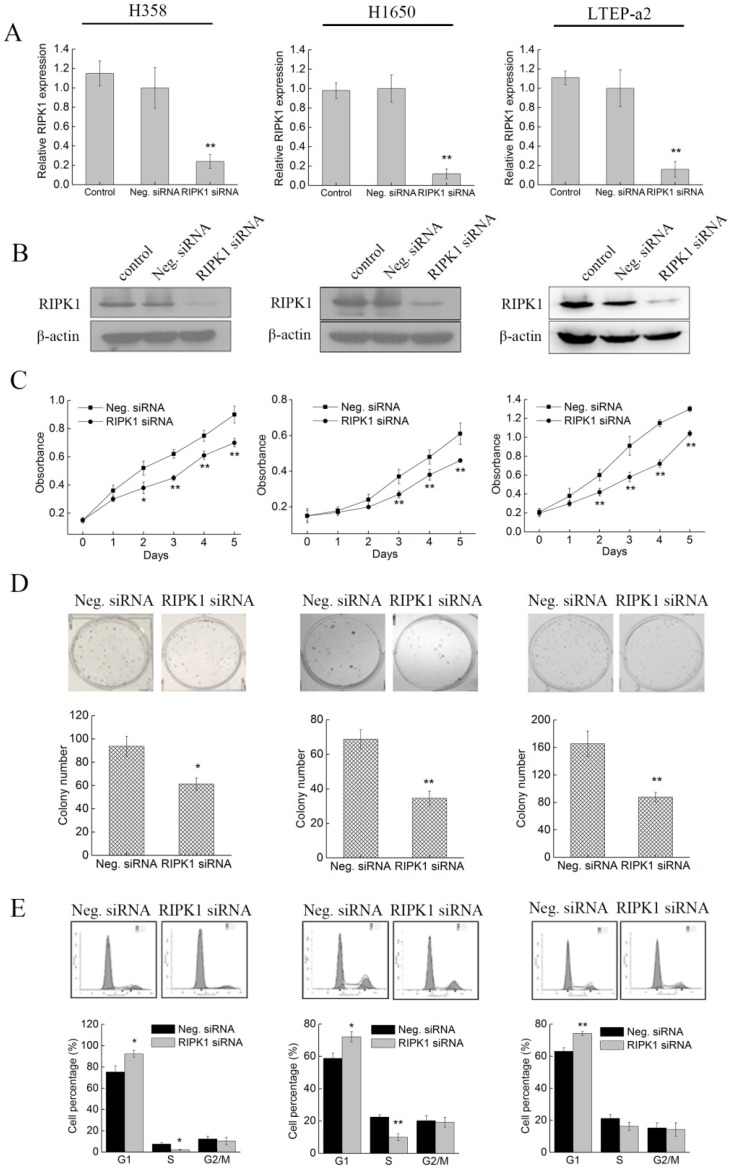
Knockdown of RIPK1 expression inhibits the in vitro proliferation of non-small-cell lung cancer cells. (**A**) The qRT-PCR analysis of RIPK1 expressions in H358, H1650, and LTEP-a2 cells after transfected with Neg. and RIPK1 siRNA. All cells were exposed to Neg. and RIPK1 siRNA for 12 h, followed by culturing with complete medium for 36 h before RNA extraction and qRT-PCR analysis. (**B**) Western blot analysis of RIPK1 expressions in H358, H1650, and LTEP-a2 cells after transfected with Neg. and RIPK1 siRNA. All cells were exposed to Neg. and RIPK1 siRNA for 12 h, followed by culturing with complete medium for 48 h before protein extraction and Western blot analysis. (**C**) Cell proliferation analysis of H358, H1650, and LTEP-a2 cells after transfection with Neg. and RIPK1 siRNA. All cells were exposed to Neg. and RIPK1 siRNA for 12 h, followed by dissociation with trypsin and transfer into 96-well plates for proliferation assay. (**D**) Cell colony formation analysis of H358, H1650, and LTEP-a2 cells after transfection with Neg. and RIPK1 siRNA. All cells were exposed to Neg. and RIPK1 siRNA for 12 h, followed by dissociation with trypsin and transfer into 6-well plates for colony formation analysis. (**E**) Cell cycle distribution analysis of H358, H1650, and LTEP-a2 cells after transfection with Neg. and RIPK1 siRNA. All cells were exposed to Neg. and RIPK1 siRNA for 12 h, followed by culturing with complete medium for 36 h before cell cycle analysis. RIPK1 = receptor-interacting serine/threonine-protein kinase 1; Neg. siRNA = negative control siRNA. Bars represent mean ± SD; * *p* < 0.05; ** *p* < 0.01.

To figure out whether phycocyanin exerted antineoplastic effects through reducing RIPK1 expressions in NSCLC cells, we performed a knockdown experiment using specific RIPK1 siRNA. The silencing effect was validated by qRT-PCR and Western blot. As shown in Figure 5A,B, siRNA transfection significantly reduced the transcription and protein levels of RIPK1 in H358, H1650, and LTEP-a2 cells, revealing RIPK1 was successfully silenced in tested cells. Later, cell growth abilities were examined after siRNA transfection. As shown in Figure 5C,D, compared with Neg. siRNA transfection groups, siRNA knockdown of RIPK1 greatly inhibited the proliferation and colony formation abilities of H358, H1650, and LTEP-a2 cells. In addition, the proportion of G1 phase cells significantly increased in RIPK1 knockdown NSCLC cells (Figure 5E), suggesting that silencing RIPK1 expression could restrain the growth of NSCLC cells through inducing G1 phase arrest. It was worth noting that these results were strongly in accordance with the proliferation phenotype in phycocyanin-treated cells, which indicated that phycocyanin exerted an anti-proliferative function through down-regulation of RIPK1 in NSCLC cells.

### 2.6. Knockdown of RIPK1 Expression Suppresses the In Vitro Migration of Non-Small-Cell Lung Cancer Cells

**Figure 6:**
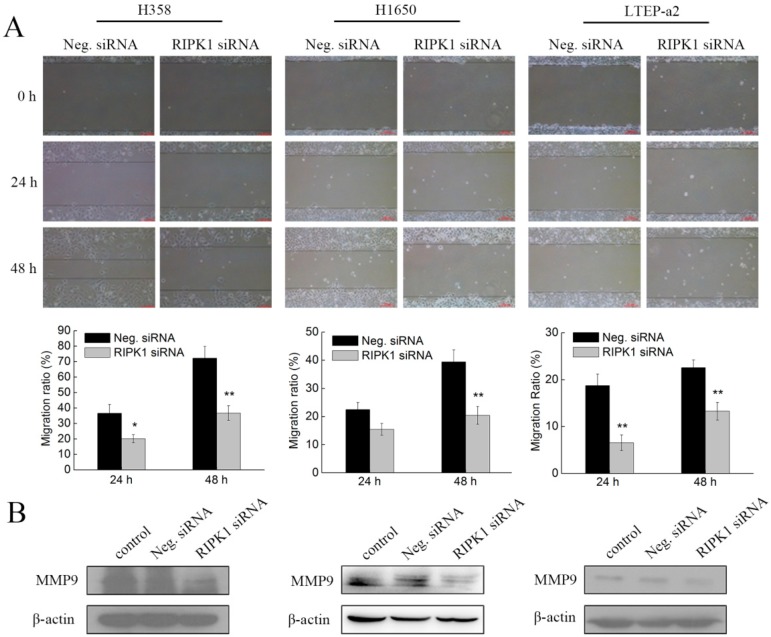
Knockdown of RIPK1 expression suppresses the in vitro migration of non-small-cell lung cancer cells. (**A**) The wound-healing assay showed representative effects of RIPK1 siRNAs transfection on H358, H1650, and LTEP-a2 cell migration at 24 and 48 h. All cells were exposed to Neg. and RIPK1 siRNA for 12 h, followed by cell migration analysis. Scale bars represent 100 µm. Quantification of wound closure was shown by histogram. (**B**) Western blot analysis of MMP9 expression in NSCLC cells after transfection with Neg. and RIPK1 siRNAs. All cells were exposed to Neg. and RIPK1 siRNA for 12 h, followed by culturing with complete medium for 48 h before protein extraction. MMP9 = matrix metalloproteinase-9. Bars represent mean ± SD; * *p* < 0.05; ** *p* < 0.01.

The effect of RIPK1 siRNA on cell migration was also determined by a wound-healing assay. As expected, in accordance with the phycocyanin-treated results, siRNA knockdown of RIPK1 expression significantly inhibited the in vitro migration of H358, H1650, and LTEP-a2 cells in a time-dependent manner (Figure 6A). As shown in Figure 6B, RIPK1 siRNA transfection also reduced the expressions of MMP9 in three tested NSCLC cells, which further supported the wound-healing analysis. Taken together, these results suggested that RIPK1 was also involved in the phycocyanin-mediated migration inhibition process in NSCLC cells.

### 2.7. Knockdown of RIPK1 Expression Induces Apoptosis of Non-Small-Cell Lung Cancer Cells

**Figure 7:**
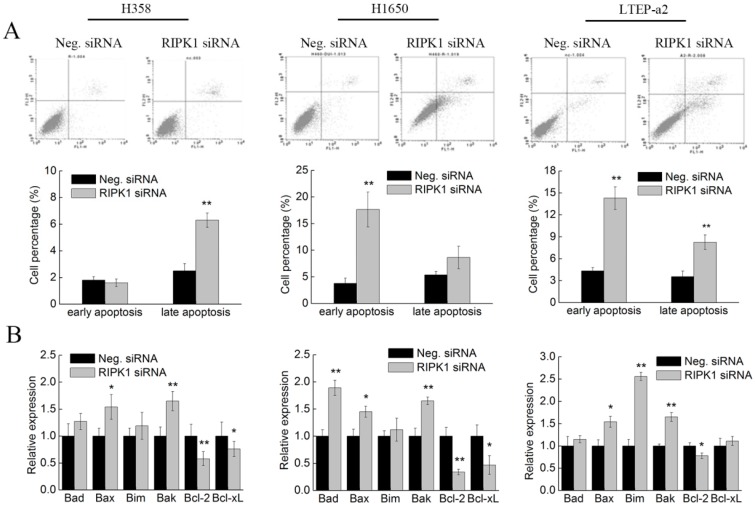
Knockdown of RIPK1 expression induces apoptosis of non-small-cell lung cancer cells. (**A**) Cell apoptosis analysis using Annexin V-fluorescein isothiocyanate (FITC)/propidium iodide (PI) staining method. Cells were transfected with Neg. and RIPK1 siRNA for 12 h, followed by culturing with complete medium for 36 h before apoptosis testing. The proportion of early and late apoptotic cells are shown by histogram. (**B**) The qRT-PCR analysis of apoptotic markers in NSCLC cells after transfection with Neg. and RIPK1 siRNA. All cells were exposed to Neg. and RIPK1 siRNA for 12 h, followed by culturing with complete medium for 48 h before RNA extraction. Bars represent mean ± SD; * *p* < 0.05; ** *p* < 0.01.

The cell apoptosis experiments were performed in RIPK1 siRNA transfected NSCLC cells. As shown in Figure 7A, with Neg. transfected groups, siRNA knockdown of RIPK1 expression greatly promoted the proportion of early apoptotic H1650 (17.37% ± 1.45%) and LTEP-a2 (13.23% ± 2.21%) cells. Meanwhile, silencing of RIPK1 expression also caused significant increases of late apoptosis cells in H358 (6.15% ± 0.23%) and LTEP-a2 (8.42% ± 1.12%) cell lines, which indicates that RIPK1 participated in the apoptotic regulatory processes in multiple NSCLC cells. Likewise, RIPK1 knockdown induced the transcriptional levels of pro-apoptotic genes Bak and Bax, in addition to reducing the expression of Bcl-2 in H358, H1650, and LTEP-a2 cells (Figure 7B). Therefore, combined with the results of Figure 3, these results suggested that RIPK1 was involved in the phycocyanin-mediated apoptosis-inducing effects in NSCLC cells.

### 2.8. Phycocyanin Inhibits the Proliferation and Migration of Non-Small-Cell Lung Cancer Cells Through Down-Regulation of RIPK1/NF-κB Activity

**Figure 8:**
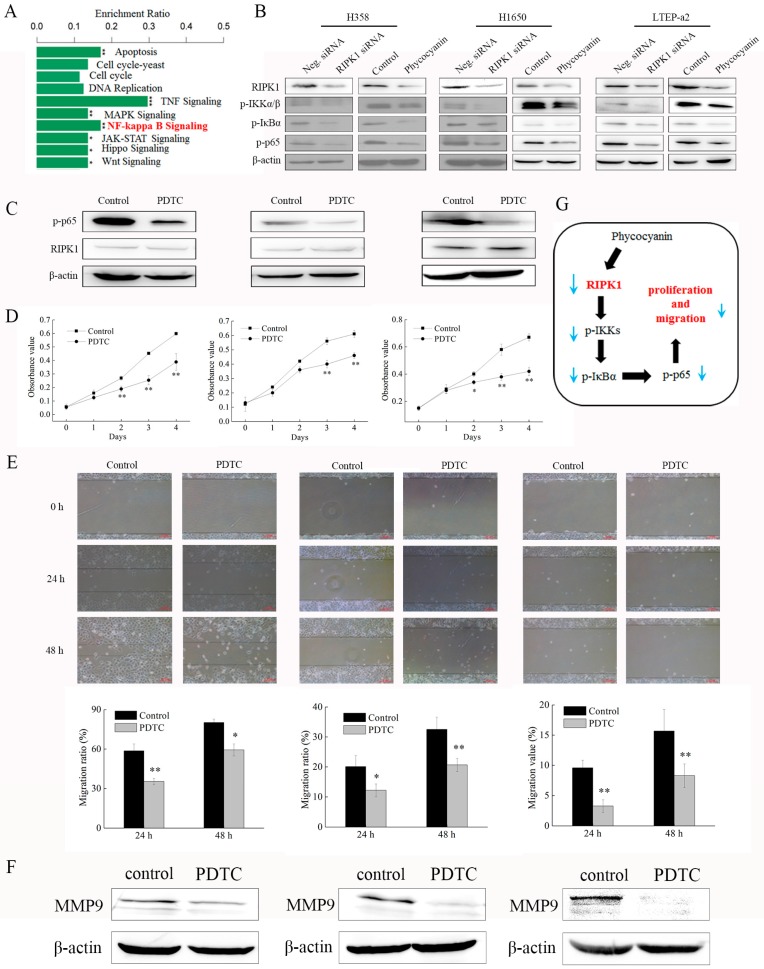
Phycocyanin inhibits the proliferation and migration of non-small-cell lung cancer cells through down-regulation of RIPK1. (**A**) RNA-seq analysis of the potential pathways involved in phycocyanin-mediated biological processes in LTEP-a2 cells. (**B**) Western blot analysis of RIPK1 and NF-κB pathway expressions in H358, H1650, and LTEP-a2 cells after treatment with RIPK1 siRNA and phycocyanin, respectively. For phycocyanin treatment, proteins were extracted after being treated with phycocyanin and control for 72 h. For siRNA transfection, cells were exposed to Neg. and RIPK1 siRNA for 12 h, followed by culturing with complete medium for 48 h before protein extraction. (**C**) Western blot analysis of RIPK1 and phosphorylated p65 expressions in H358, H1650, and LTEP-a2 cells at 72 h after treatment with 10 µM PDTC for 24 h. (**D**) Cell proliferation analysis of H358, H1650, and LTEP-a2 cells after treatment with 10 µM PDTC for 24 h. (**E**) Wound-healing analysis of H358, H1650, and LTEP-a2 cells after treatment with 10 µM PDTC for 24 h. (**F**) Western blot analysis of MMP9 expression in NSCLC cells after PDTC treatment. (**G**) Illustration of phycocyanin/RIPK1/NF-κB regulation process in NSCLC cells. PDTC = pyrrolidine dithiocarbamate. Bars represent mean ± SD; * *p* < 0.05; ** *p* < 0.01.

The transcriptome results indicated that NF-κB signaling activity was significantly reduced after phycocyanin treatment (Figure 8A). On the basis of RNA-seq results, the effects of phycocyanin and RIPK1 on NF-κB activity in NSCLC cells were examined. As shown in Figure 8B, besides RIPK1, phycocyanin decreased the phosphorylation levels of IKKα/β, IκBα, and p65 in H358, H1650, and LTEP-a2 cells, which was in accordance with Bingula’s research on A549 cell lines [26]. In addition, siRNA knockdown of RIPK1 expression also inhibited the phosphorylation levels of these proteins, revealing that RIPK1 had regulatory function on NF-κB activity in NSCLC cells. To confirm the above results, we also examined the total expressions and phospho/total ratios of IKKα/β, IκBα, and p65 proteins (Figure S3). As expected, RIPK1 siRNA and phycocyanin treatment could significantly decrease the phospho/total ratios of IKKα/β, IκBα, and p65 in three cell lines, which further indicated a reduced activity of NF-κB signaling. To figure out whether phycocyanin exerted anti-proliferative and anti-migratory effects on NSCLC cells through the RIPK1/NF-κB pathway, we employed a pyrrolidine dithiocarbamate (PDTC) treatment experiment. PDTC is a classical NF-κB inhibitor, which could suppress the phosphorylation of IKKα/β and p65 [34]. As expected, blockage of NF-κB activity with PDTC significantly inhibited the in vitro proliferation and migration of H358, H1650, and LTEP-a2 cells (Figure 8C–E). Low concentration of PDTC (2.5 µM) also decreased the migration ability of NSCLC cells, despite the fact that the proliferation was not affected (Figure S4). In addition, the expressions of MMP9 were also decreased after PDTC exposure (Figure 8F). Strikingly, PDTC treatment had no effect on RIPK1 expression in tested cells, indicating that RIPK1 is located upstream of NF-κB pathway (Figure 8C). Taken together, the illustration of the potential anticancer mechanism of phycocyanin in NSCLC cells is presented in Figure 8G. 

## 3. Discussion

The anticancer function of phycocyanin in NSCLC has been investigated in different studies. Czerwonka et al. reported that Spirulina extract could cause G1 phase arrest and Bax:Bcl-2 ratio increase in NSCLC A549 cells [35], which was similar to our results (Figure 1 and Figure 3). Strikingly, Spirulina extract only contained 12–19% phycocyanin, which from one side reflected the antineoplastic effect of Spirulina. It is worth noting that Huang et al. explored the anticancer effect of phycocyanin using a phycocyanin-based nanocarrier as a novel platform, promoting the antineoplastic efficacy in vivo and in vitro [36]. Likewise, Deniz and Thangam et al. demonstrated the prevention activity of phycocyanin against highly lethal lung cancer using a A549 cell model as well [37,38]. In addition, besides phycocyanin, phycoerythrin, which is also derived from cyanobacteria, has been proven to exert pro-apoptotic and anti-proliferative function in A549 cells [39,40], suggesting the potential pharmaceutical value of phycobiliproteins. Overall, the A549 cell line has been widely used in multiple investigations on functional materials; nevertheless, our study employed H358, H1650; and LTEP-a2 NSCLC cell lines to explore the antineoplastic and underlying mechanism of phycocyanin, which has widely illustrated the biological activities of phycocyanin in NSCLC cells.

Nuclear factor-κB (NF-κB) consists of a family of transcription factors playing critical roles in various biological processes, including inflammation, immunity, cell proliferation, differentiation, and survival [41]. Phyoccyanin was reported to participate in the regulation of multiple physiological activities through the NF-κB pathway. For example, Zhu et al. and Cherng et al. reported that selenium-containing phycocyanin could reduce inflammation in dextran sulfate sodium-induced colitis via inhibiting NF-κB activity in vivo and in vitro, respectively [42,43]. Coincidentally, our previous study had confirmed their results by using a lipopolysaccharide-induced RAW 264.7 macrophages model [44]. It was found that the TLR2-MyD88-NF-κB pathway played an important role in phycocyanin-mediated reduction in lung injuries [45,46]. Particularly, NF-κB activity was greatly reduced by phycocyanin, which was in accordance with our results (Figure 8B). In fact, the role of NF-κB in human cancer initiation, metastasis, development, and resistance to treatment has drawn particular attention in recent years [47]. Phycocyanin was found to exert an antineoplastic effect on liver and pancreas cancer through reducing NF-κB activity [17,48]. In addition, Bingula et al. discovered a reduced expression of NF-κB in phycocyanin-treated A549 cell line [26], which was also reported in our previous work [49]. Overall, recent studies have demonstrated that phycocyanin could act as a NF-κB suppressor in different tumor cells, including NSCLC A549 cells. Nevertheless, the present work revealed a potential NF-κB regulatory factor, RIPK1, involved in phycocyanin-mediated anticancer process, which suggests a novel underlying modulating mechanism of phycocyanin in NSCLC cell lines. These results undoubtedly provide a significant theoretical basis to explore the regulation mechanism of phycocyanin in NSCLC.

The kinase RIPK1 is an essential signaling node in various physiological processes, including innate immune regulation, cell growth, cell death, and so on [33,50]. In this study, knockdown of RIPK1 expression resulted in the reduced proliferation and migration of H358, H1650, and LTEP-a2 cells (Figure 5 and Figure 6), indicating RIPK1 participated in regulating proliferation and migration of NSCLC cell lines. As a matter of fact, aberrant expression of RIPK1 could lead to the development of multiple tumors, including liver cancer [51], pancreatic cancer [52], melanoma [53], and so on. Interestingly, Van et al discovered that ablating RIPK1 in liver parenchymal cells did not cause spontaneous liver pathology, while combined deficiency of RIPK1 and RelA protein would lead to chronic liver disease and hepatocellular carcinoma [54]. By contrast, our study revealed that aberrant expression of RIPK1 alone could influence the proliferation, migration, and apoptosis of NSCLC cell lines (Figure 5, Figure 6 and Figure 7), which might indicate different regulatory mechanisms of RIPK1 in different cells. Strikingly, Jing et al. discovered that RIPK1 played a key role in modulating cisplatin-triggered cell death in lung cancer A549 cells [55], which partly supported our results. However, these have been no articles that have reported the regulation relationship between phycocyanin and RIPK1 in NSCLC cells yet. In this present study, RIPK1 was identified as an important factor that took part in the phycocyanin-mediated proliferation and migration inhibition processes in H358, H1650, and LTEP-a2 cell lines. To the best of our knowledge, this is the first investigation on the correlation of RIPK1 and phycocyanin in NSCLC. Although the precise regulatory mechanism between phycocyanin and RIPK1 still needs further exploration, our work undoubtedly lays a theoretical basis on the potential treatment of NSCLC with marine functional products.

## 4. Materials and Methods 

### 4.1. Materials, Cell Lines and Culture Conditions

Phycocyanin (extracted from Spirulina platensis, >90% purity) standard substance was purchased from Envirologix (Portland, ME, USA). Phycocyanin was dissolved in phosphate buffer solution (PBS) according to the specification. Human NSCLC H358, H1650, and LTEP-a2 cell lines were purchased from American Type Cell Collection (ATCC, Manassas, VA, USA). Cells were cultured in DMEM media supplemented with 10% fetal bovine serum (FBS) at 37 °C in a humidified atmosphere with 5% CO2. Cells were sub-cultured every 2–3 days. Cells between 3–10 passages were used in the experiments. 

### 4.2. siRNA Transfection

The siRNA transfection was performed as described in our previous work [56]. Briefly, cells were seeded into a 6-well plate with an appropriate density beforehand, followed by transfection with 80 nM of a siRNA (GenePharma, Shanghai, China) for each well using Dhama FECT 1reagent according to the manufacturer’s instructions (Dharmacon, Lafayette, CO, USA). Negative siRNA was used as the negative control. The cells were exposed to siRNA and negative control for 12 h, followed by replacement of the medium before proceeding with subsequent experiments. 

### 4.3. Cell Morphology Observation

Cells were seeded at an appropriate density into 6-well plates. After attachment, phycocyanin was added into each well for 48 h, followed by morphology observation using optical microscopy (Olympus, Japan). For siRNA transfected cells, a similar process was performed as described above.

### 4.4. Cell Proliferation Assay

Cell proliferation was determined by 3-(4,5-dimethyl-2-thiazolyl)-2,5-diphenyl-2-H-tetrazolium bromide (MTT) method, as described in our former study [57]. Briefly, after incubation with 7.5 µM phycocyanin for 24 h, cells were seeded at an appropriate density into 96-well plates the day before detection. The NSCLC cells were incubated with MTT for 4 h, followed by Sodium Dodecyl Sulfonate (SDS)-HCl solution addition each day. The absorbance was detected at 570 and 630 nm. The proliferation assay lasted for 5 or 6 days. Three independent experiments were carried out. For the proliferation analysis of siRNA transfected cells, cells were exposed to siRNA and negative control for 12 h, followed by dissociation with trypsin and transfer into 96-well plates. A similar process was performed as described above.

### 4.5. Cell Viability Assay

Cell viability was determined by MTT method, as described in our former work [19]. Briefly, cells were seeded at an appropriate density into 96-well plates the day before phycocyanin addition. Then, cells were incubated with phycocyanin at indicated final dosages (0, 2.5, 5, 7.5, and 10 µM) for 24 h. After incubation, MTT was added into each well for 4 h, followed by dimethylsulfoxide (DMSO) dissolution. The absorbance was measured at 630 and 460 nm. The cell viability was shown as the ratio of the absorbance reading and its corresponding control cells.

### 4.6. Cell Colony Formation Assay

Cells were seeded at about 300 cells per cell in 6-well plates. After 12 h incubation, cells were treated for another 24 h with appropriated phycocyanin, followed by continuous incubation in fresh medium at 37 °C in a cell incubator. After 12–15 days, cells were washed with PBS, fixed with methanol for 15 min, and stained with 0.5% crystal violet for 15 min at room temperature. The number of cell colonies was counted for analysis. For cells with siRNA transfection, cells were exposed to siRNA and negative control for 12 h, followed by dissociation with trypsin and transfer into 6-well plates. A similar process was performed as described above.

### 4.7. Cell Cycle Assay

After treatment with phycocyanin for 48 h, cells were harvested and fixed in 1 mL 70% cold ethanol and incubated at 4 °C for at least 48 h. Cells were centrifuged at 1500 rpm for 5 min and resuspended in 500 µL of propidium iodide (PI)/RNase staining buffer, followed by incubation on ice for 30 min. The cells were washed twice with cold PBS before detection. Cell cycle distribution was measured using FACSCalibur (Becton Dicknson, Franklin Lakes, NJ, USA). For cells with siRNA transfection, cells were exposed to siRNA and negative control for 12 h, followed by culturing with complete medium for 36 h before harvest. Cell collection and fixing processes were performed as described above.

### 4.8. Wound-Healing Assay

Cells in the exponential growth phase were seeded in 6-well plates. After attachment, cell were treated for phycocyanin, and then continuously incubated in fresh medium (containing 3% fetal bovine serum) at 37 °C in 5% humidified CO2. The culture insert provided two cell culture reservoirs that were separated by a thick wall. A “wound” was formed between the two cell patches. Photos of the wounds were taken every 12 h. The widths of the wounds were measured at three positions for each replicate using Leica Application Suite (Leica Microsystems GmbH, Wetzlar, Germany). For cells with siRNA transfection, cells were exposed to siRNA and negative control for 12 h, followed by wound-healing analysis. A similar process was performed as described above.

### 4.9. Cell Apoptosis Assay

After treated with phycocyanin for 48 h, cells were harvested and washed twice with cold PBS and then resuspended in 500 µL binding buffer. Then, cells were stained in 5 µL Annexin V-FITC/PI according to the manufacturer’s protocol (Roche, Mannheim, Germany). Stained cells were analyzed by FACSCalibur (Becton Dickson). For cells with siRNA transfection, cells were exposed to siRNA and negative control for 12 h, followed by culturing with complete medium for 36 h before harvest. Cell collection and staining processes were performed as described above.

### 4.10. Transcriptome Analysis

LTEP-a2 cells were selected for RNA-seq analysis using Illumina HiSeq 4000 (Illumina, San Diego, CA, USA). Briefly, after treatment with phycocyanin, total RNA was extracted using Trizol regent (Invitrogen, Carlsbad, CA, USA). Five µg of total RNA was used for analysis. Base calling was adopted to convert original sequencing images to sequential data. Human genome sequence and gene annotation were obtained from the University of California, Santa Cruz (UCSC) Genome Website. The differentially expressed genes (DEGs) between phycocyanin-treated and control cells were identified based on fragments per kilobases per million reads (FPKM) using RNA-Seq by Expectation-Maximization (RSEM) 1.2.31 [58]. DESeq was used to determine the adjusted p value. If the adjusted p value was less than 0.05, it was considered to be a significantly different expression level.

### 4.11. Quantitative RT-PCR (qRT-PCR)

After being treated with 7.5 µM phycocyanin for 48 h, cells were collected for RNA extraction. Total RNA was extracted using Trizol reagent and reverse-transcribed with PrimeScript RT Master Mix. Real-time PCR analysis was performed in an Applied Biosystems Step One-Plus (Waltham, MA, USA). The relative expression of each targeted gene was calculated and normalized using 2-ΔΔCt method relative to reduced glyceraldehyde-phosphate dehydrogenase (GAPDH). For siRNA transfection, cells were exposed to siRNA and negative control for 12 h, followed by culturing with complete medium for 36 h before harvest and RNA extraction. The primers used in qRT-PCR were shown in Table S1. Each assay was performed in quadruplicate.

### 4.12. Western Blot Analysis

After being treated with 7.5 µM phycocyanin for 72 h, cells were collected for protein extraction. Proteins were extracted by RIPA lysis buffer (1% NP40, 0.1% SDS, 5 mM EDTA, 0.5% sodium deoxycholate, 1 mM sodium orthovanadate) containing protease and phosphatase inhibitors. Equivalent amounts of proteins were separated by 12% sodium dodecyl sulfate polyacrylamide gel electrophoresis (SDS-PAGE), and then electro-transferred onto polyvinylidene fluoride (PVDF) membranes. After blocking with 5% skim milk, the membranes were incubated with primary antibodies at 4 °C overnight, followed by incubation with horseradish peroxidase-conjugated secondary antibodies. For siRNA transfection, cells were exposed to siRNA and negative control for 12 h, followed by culturing with complete medium for 48 h before harvest and protein extraction. Signals were detected by an electrochemiluminescence (ECL) system.

### 4.13. Statistical Analysis

The numerical data were expressed as means ± SD. Two-tailed student’s t-test was performed for comparison among the different groups. In addition, *p* < 0.05 (*) or *p* < 0.01 (**) was considered as statistically significant.

## Supplementary Materials

The following are available online at https://www.mdpi.com/1660-3397/17/6/362/s1, Supplementary Table S1. Primers used for qRT-PCR. Figure S1. Cell survival rate analysis of NSCLC cells after phycocyanin treatment. Figure S2. Proliferation and migration analysis of NSCLC cells after treatment with low concentration of phycocyanin (2.5 µM). Figure S3. Western blot analysis of total expressions of IKKα/β, IκBα, p65, and phosphor/total ratios analysis of these proteins. Figure S4. Proliferation and migration analysis of NSCLC cells after treatment with low concentration of PDTC (2.5 µM).
