# Supplementary material for: C-Phycocyanin Suppresses the In Vitro Proliferation and Migration of Non-Small-Cell Lung Cancer Cells through Reduction of RIPK1/NF-κB Activity
Source: Mar Drugs. 2019 Jun 18;17(6):362. doi: 10.3390/md17060362 (PMC6627888; doi:10.3390/md17060362)
Supplement: Supplementary file 1 [file marinedrugs-17-00362-s001.zip › Supplementary Material.pdf]

Article

C-phycocyanin suppresses the in vitro proliferation and migration of non-small cell lung cancer cells through reducing RIPK1/NF-κB activity

Shuai Hao *, Shuang Li, Jing Wang, Lei Zhao, Yan Yan, Tingting Wu, Jiawen Zhang and Chengtao Wang *

Beijing Advanced Innovation Center for Food Nutrition and Human Health, Beijing Engineering and Technology Research Center of Food Additives, Beijing Technology and Business University, Beijing 100048, China; lishuangldw@163.com (S.L.); trotwj960@163.com (J.W.); zhaolei@th.btbu.edu.cn (L.Z.); 15128470659@163.com (Y.Y.); m18810529269@163.com (T.W.); zhangjiawen98@outlook.com (J.Z.)

* Correspondence: Correspondence: haoshuai@btbu.edu.cn (S.H.); ctwangbtbu@163.com (C.W.)

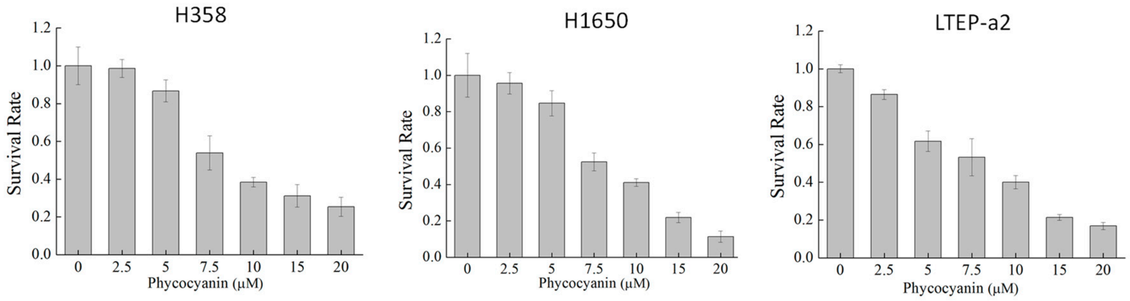

Figure S1. Cell survival rate analysis of NSCLC cells after phycocyanin treatment. H358, H1650 and LTEP-a2 cell lines were treated with different concentrations (0, 2.5, 5, 7.5, 10, 15, and 20 μM) of phycocyanin for 48 h and subjected to a survival rate analysis.

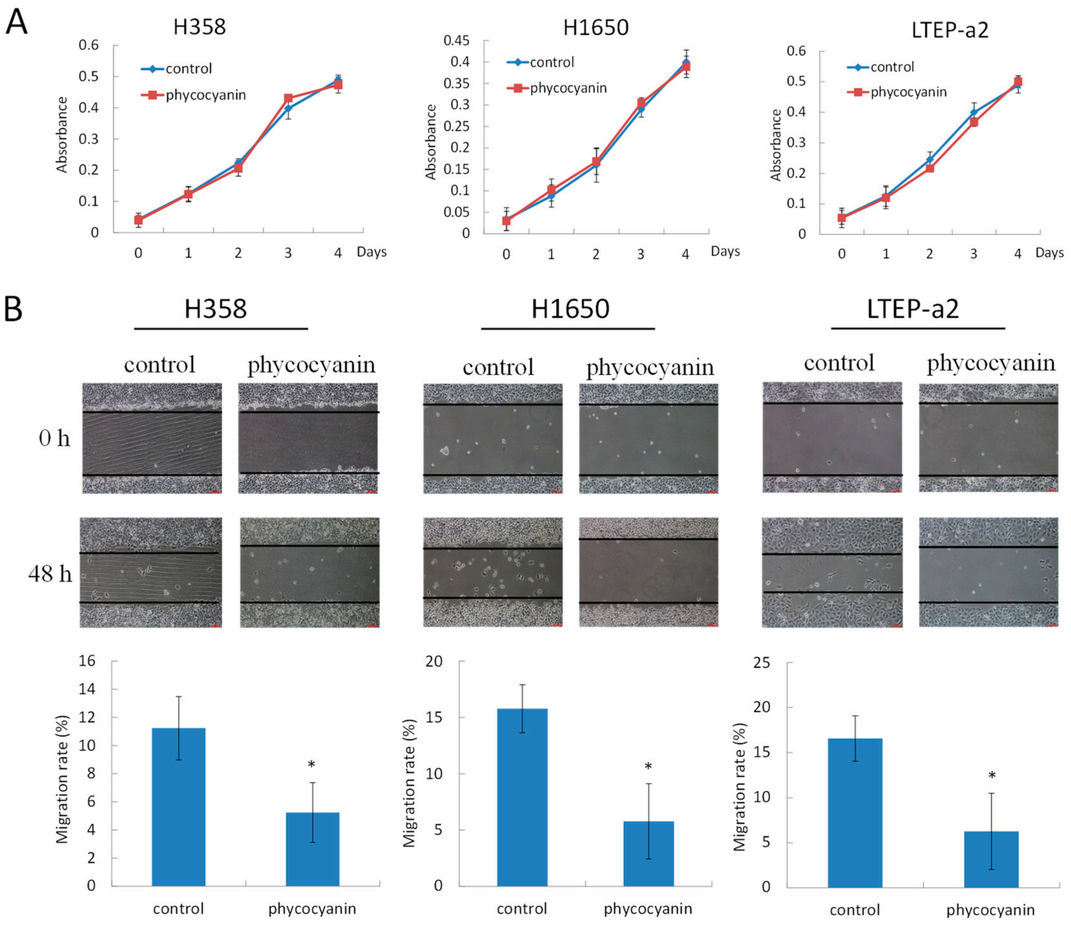

Figure S2. Proliferation and migration analysis of NSCLC cells after treatment with low concentration of phycocyanin (2.5 μM). (A) Proliferation analysis of NSCLC cells after 2.5 μM phycocyanin treatment. (B) Wound-healing analysis of the migration of NSCLC cells after 2.5 μM phycocyanin treatment. Bars represent mean ± SD. *, p<0.05; **, p<0.01.

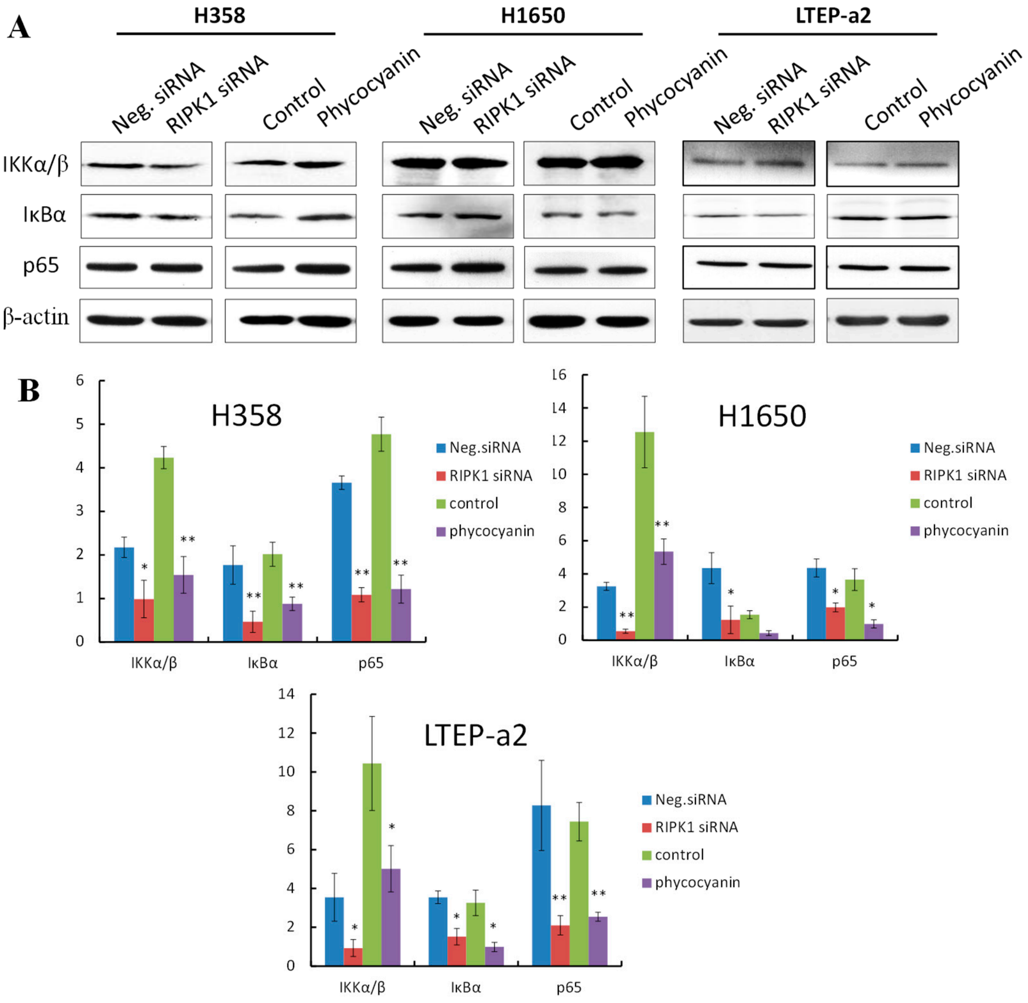

Figure S3. Western blot analysis of total expressions of IKKα/β, IκBα, p65 and phosphor/total ratios analysis of these proteins. (A) Western blot analysis of total expressions of IKKα/β, IκBα and p65 proteins after RIPK1 siRNA and phycocyanin treatment, respectively. (B) Phospho/total ratios of these proteins in three NSCLC cell lines. Bars represent mean ± SD. *, $p<0.05$; **, $p<0.01$.

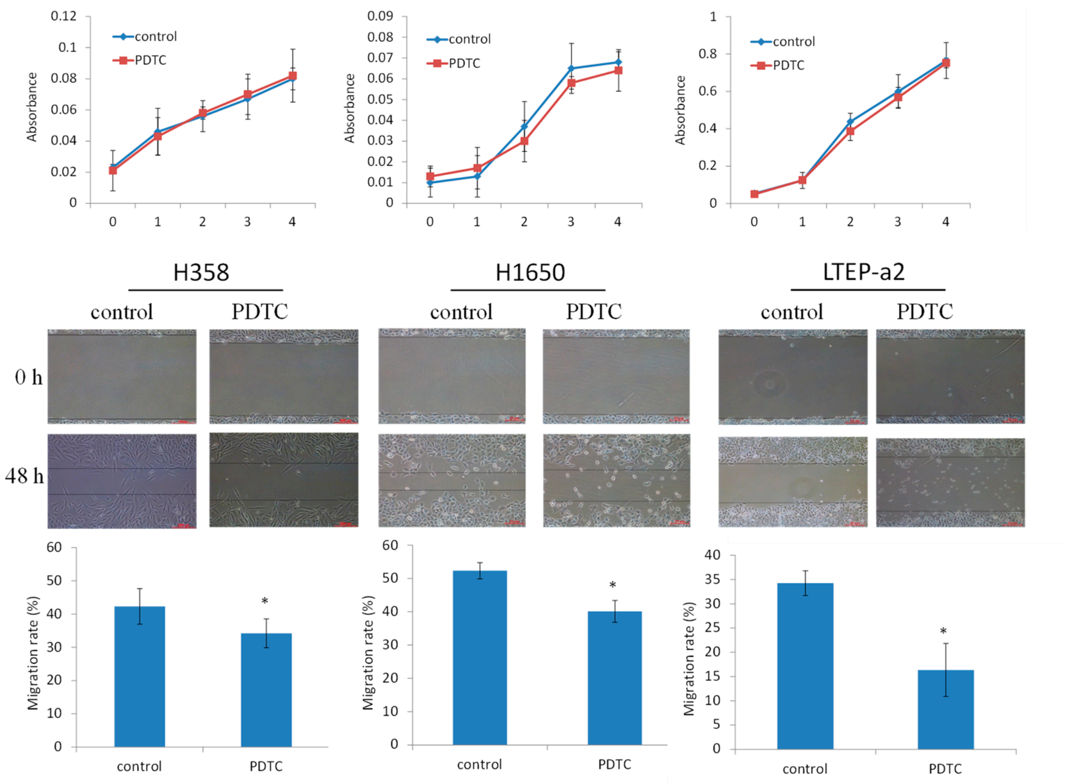

Figure S4. Proliferation and migration analysis of NSCLC cells after treatment with low concentration of PDTC (2.5 μM). (A) Proliferation analysis of NSCLC cells after 2.5 μM PDTC treatment. (B) Wound-healing analysis of the migration of NSCLC cells after 2.5 μM PDTC treatment. Bars represent mean ± SD. *, $p<0.05$; **, $p<0.01$.
